# Experimental Investigation and Predictive Modeling of Cumulative Plastic Deformation of Silty Sand Under Freeze–Thaw Cycles and Cyclic Loading

**DOI:** 10.3390/ma19122461

**Published:** 2026-06-09

**Authors:** Dongkai Ma, Zhongming He, Yiwei Li, Zhenhong Yan, Chao Huang

**Affiliations:** 1 School of Traffic and Transportation on Engineering, Changsha University of Science & Technology, Changsha 410004, China; 2 National Engineering Research Center of Highway Maintenance Technology, Changsha 410004, China; 3 School of Civil Engineering, Central South University, Changsha 410075, China

**Keywords:** freeze–thaw cycles, silty sand, cumulative plastic deformation, prediction model

## Abstract

The long-term deformation and stability of silty sand roadbeds subjected to repeated freeze–thaw cycles and traffic loading remain ongoing engineering concerns in seasonally frozen regions. To investigate the evolution and influencing factors of accumulative axial plastic deformation of silty sand under freeze–thaw cycles, this study focused on silty sand from a roadbed construction site in Inner Mongolia, China, a typical seasonally frozen region. Dynamic triaxial tests were conducted under loading stresses of 60–100 kPa, confining pressures of 20–60 kPa, water contents ranging from OMC to 1.2 OMC, and freeze–thaw cycles of 0–10. The results indicate that approximately 60–80% of the total accumulative axial plastic deformation occurs within the first 1000 loading cycles, after which the deformation growth rate gradually decreases. Increases in loading stress, water content, and freeze–thaw cycles promote deformation, whereas higher confining pressures suppress it. For example, increasing the confining pressure from 20 to 60 kPa reduced the final deformation from 0.16% to 0.07%, while increasing the number of freeze–thaw cycles from 0 to 10 increased the final deformation from 0.10% to 0.28%. Based on the experimental data, a new predictive model considering net stress, octahedral shear stress, water content ratio, and freeze–thaw cycles was developed. The model demonstrates high accuracy in predicting accumulative plastic deformation, with a coefficient of determination of 0.915, and is applicable to both plastically stable and weakly plastic creep conditions. This study provides a reference for the design, construction, and mitigation of subgrade damage in silty sand roadbeds in seasonally frozen regions.

## 1. Introduction

China has extensive permafrost and seasonally frozen ground, with seasonally frozen soil covering approximately 53.5% of the national territory [1], mainly distributed in North China, Northeast China, Inner Mongolia, and the Qinghai–Tibet Plateau. In these regions, subgrade heave and thaw settlement caused by seasonal freeze–thaw cycles have become significant engineering challenges for transportation infrastructure [2]. Engineering practice has shown that silty sand is commonly encountered in subgrade construction in seasonally frozen regions. In China, silty sand may be used as subgrade fill when it satisfies the requirements for compaction quality, bearing capacity, moisture condition, and relevant design specifications. However, for roadbed zones in seasonally frozen regions or water-affected sections, the direct use of silty or fine-grained soils may be restricted, and stabilization, improvement, or drainage measures may be required [3]. Due to its strong capillary action, high water absorption, and water retention capacity [4], silty sand may be sensitive to freeze–thaw cycles and repeated traffic loading. Under repeated freeze–thaw cycles, such materials may experience increased deformation and settlement risk if moisture and drainage conditions are not properly controlled, which can adversely affect pavement serviceability [5]. Therefore, to improve the safety and durability of roads in seasonally frozen regions under freeze–thaw conditions, it is particularly important to investigate the deformation and damage characteristics of silty sand under cyclic freezing and thawing.

Cumulative plastic deformation is a key indicator for evaluating the long-term stability of subgrade soils under repeated traffic loading, particularly in seasonally frozen regions where traffic loading, moisture variation, freeze–thaw action, and confinement act simultaneously. Previous studies have shown that the stress state strongly affects deformation accumulation. Monismith et al. [6] pointed out that the stress loading path plays an important role in the evolution of accumulative deformation. Xu et al. [7] found through dynamic triaxial tests on frozen soil that confining pressure suppresses axial deformation. Zheng et al. [8] also reported that, compared with freezing temperature, freeze–thaw cycles and stress conditions have more pronounced effects on the mechanical behavior of remolded loess. Field monitoring by Meng et al. [9] showed that freeze–thaw cycles can significantly amplify the effect of traffic loading, with deformation during the thawing period being particularly pronounced. Feng et al. [8] further indicated that cumulative plastic deformation is positively correlated with the number of freeze–thaw cycles, and that the most significant increase generally occurs during the initial cycles. In addition, the confining pressure level has been recognized as an important factor controlling freeze–thaw deterioration. Chen et al. [10] investigated the physical and mechanical deterioration of sandstone subjected to freeze–thaw cycles under low confining pressure and found that freeze–thaw cycling caused strength degradation and internal damage. Zhang et al. [11] further reported that freeze–thaw cycles promote the development of microdefects in rock, whereas higher confining pressure can close some microcracks and reduce freeze–thaw-induced damage. Similar deterioration has also been observed in frozen–thawed soils. Liu et al. [12] found that freeze–thaw cycles significantly changed the mechanical properties of silty sand, while studies on silty clay and loess showed that confining pressure affects the stress–strain behavior, strength deterioration, and failure mode of soils after freeze–thaw cycles [13,14]. These findings indicate that low confining pressure may provide insufficient lateral restraint, thereby aggravating freeze–thaw-induced structural damage and deformation accumulation in shallow subgrade soils. Therefore, under the combined effect of multiple factors, conducting research on the cumulative plastic deformation law of silty sand under freeze–thaw cycles and long-term traffic load is meaningful for the design and construction of sandy soil roadbeds.

In studies on accumulative deformation, the critical dynamic stress identification method based on stability theory has been widely used to distinguish between plastically stable, creep, and failure states [15], providing a foundation for subsequent analyses. Based on experimental results, many scholars have proposed various methods for predicting accumulative deformation, building upon classical models. In the early research, Karg et al. [16] investigated the cumulative residual strain of Belgian fine sand and adopted the empirical formula proposed by Wichtmann et al. [17], in which the effects of void ratio and loading frequency were considered. Monismith [6] conducted high-cycle repeated loading tests on silty clay and proposed an exponential model to describe the accumulation of plastic deformation with loading cycles. Puppala [18] analyzed test data from fine soil, clay, and sand and found that shear action had a significant influence on the cumulative plastic deformation of subgrade soils. Based on the Monismith model, mean normal stress and octahedral shear stress were further introduced to characterize confinement and shear effects. On the basis of Monismith’s model, they introduced mean normal stress and octahedral shear stress parameters and proposed a prediction calculation model that simultaneously characterizes the effects of confinement and shear; Chow et al. [19], based on the Mohr–Coulomb strength theory, established a model in which the ratio of shear stress induced by external loading to the ultimate shear strength of the subgrade soil is used to characterize the degree of soil damage. Later, Tang et al. [20], building on the classical logarithmic model, proposed an improved semi-logarithmic model for saturated sandy silt, which more accurately captures the evolution of dynamic permanent axial strain. Liu et al. [21] developed a predictive model for accumulative plastic strain using fractional calculus to describe the deformation evolution of aeolian soils under cyclic loading in freeze–thaw conditions, significantly improving the model’s ability to represent complex coupled effects. Sun et al. [22] applied a particle swarm optimization–backpropagation (PSO-BP) approach to construct a predictive model for clay accumulative plastic strain, achieving a prediction accuracy of up to 97%. Overall, previous studies have provided substantial insight into the prediction of accumulative plastic deformation in subgrade soils. Nevertheless, existing models have limitations: semi-logarithmic and exponential models consider only the influence of loading cycles; Chow’s model [19] incorporates material properties and stress state but does not account for environmental variations. Most models focus primarily on intrinsic material characteristics and the mechanical effects of external loads, with few addressing the influence of environmental factors. Therefore, while previous studies have investigated accumulative plastic deformation of subgrade soils, the combined effects of loading stress, confining pressure, water content, and freeze–thaw cycles on silty sand remain less comprehensively characterized. In this study, we systematically examine these factors and develop a prediction model that integrates both mechanical and environmental influences to describe the deformation behavior under realistic conditions.

This study focuses on silty sand as the research material. Freeze–thaw tests and dynamic triaxial tests were conducted on specimens with different water contents to analyze the patterns of accumulative plastic deformation of silty sand under varying stress states, moisture conditions, and numbers of freeze–thaw cycles. Based on the experimental results, an appropriate predictive model for accumulative plastic deformation was developed to provide a reference for the prediction of subgrade deformation and the mitigation of associated damage in silty sand roadbeds.

## 2. Materials and Experimental Program

### 2.1. Materials

**Figure 1:**
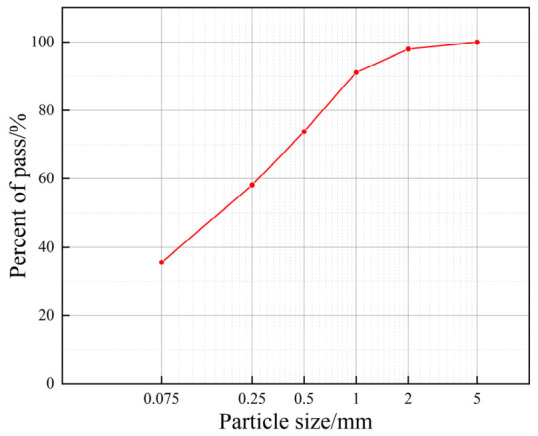
Particle size distribution curve of silty sand.

The silty sand used in this study was obtained from the subgrade construction of the Hohhot Expressway in Inner Mongolia. The basic physical properties of the soil samples were determined through particle size analysis, Atterberg limits tests, compaction tests, and CBR tests, which were conducted with reference to the Specifications for Highway Geotechnical Tests (JTG 3430-2020) [23]. The maximum dry density of the silty sand was 1.81 g/cm^3^, and the optimum moisture content was 14.9%. The liquid limit was 37.85% and the plastic limit was 25.75%, resulting in a plasticity index of 12.1. The CBR value of the silty sand at 94% compaction was 27.1%. Previous studies have also investigated the CBR characteristics of silty sand. Bąk and Chmielewski [24] reported that the measured CBR values of silty sand were approximately 12–25%, depending on the fine fraction content. Compared with these reported values, the tested silty sand in this study exhibited a relatively high bearing capacity under the specified compaction condition. In China, CBR is commonly used to evaluate the bearing capacity and suitability of subgrade filling materials. The measured CBR value also satisfies the requirement of not less than 8% specified in the Technical Specifications for Construction of Highway Subgrades (JTG 3610-2019) [25], indicating that the tested silty sand is suitable for use as subgrade fill under the specified compaction condition. The particle size distribution curve of the silty sand is shown in Figure 1.

### 2.2. Experimental Procedure

In this study, freeze–thaw tests and mechanical property investigations of silty sand were conducted using a constant-temperature and constant-humidity test chamber at the National Engineering Research Center of Highway Maintenance Technology, Changsha University of Science & Technology, along with the Dynatriax100/14 automated triaxial testing system made by the Italian Controls company (Milan, Italy). According to the Specifications for Highway Geotechnical Tests (JTG 3430-2020), the test water contents were selected as OMC (optimum moisture content), 1.1 OMC, and 1.2 OMC, rather than under fully saturated conditions. Soil specimens were prepared based on the target moisture content, sealed in plastic bags for ensuring uniform moisture distribution, and subsequently molded into cylindrical specimens using a layered static compaction method with a hydraulic press. The prepared specimens had a height of 200 mm and a diameter of 100 mm.

The prepared soil specimens were subjected to freeze–thaw cycles in a constant-temperature and constant-humidity environmental chamber. To ensure the specimens fully experienced the freeze–thaw process, the minimum freezing temperature was set to −20 °C based on temperature statistics for seasonally frozen regions [9], where subgrades commonly freeze between 0 °C and −20 °C. According to previous studies [26], each freeze–thaw cycle consisted of a 12 h freezing stage and a 12 h thawing stage, with the thawing temperature set at 20 °C. One freezing–thawing sequence constitutes a single freeze–thaw cycle, and this process was repeated until the target number of cycles was reached. Following prior research on freeze–thaw cycles [27], the number of freeze–thaw cycles (*N_FT_*) was set using an incrementally increasing scheme: *N_FT_* = 0, *N_FT_* = 1, *N_FT_* = 3, *N_FT_* = 6, and *N_FT_* = 10.

**Figure 2:**
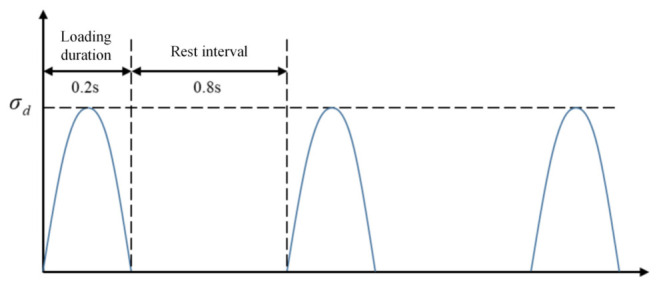
Schematic diagram of intermittent half-sine pulse loading.

**Figure 3:**
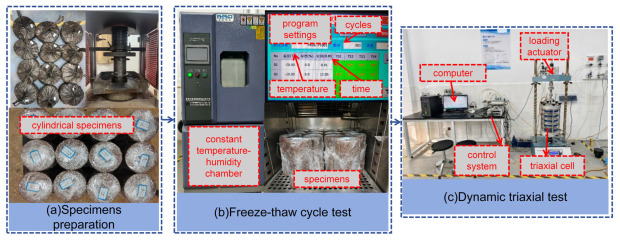
The overall experimental procedure: (**a**) specimen preparation; (**b**) freeze–thaw cycle test; (**c**) dynamic triaxial test.

The design of the dynamic triaxial testing program was determined by comprehensively considering existing research findings and the actual stress characteristics of subgrade soils. In this study, the dynamic triaxial tests were conducted under unconsolidated undrained conditions. Regarding confining pressure, subgrade confining pressure increases with depth, and previous studies indicate that it generally ranges from 0 to 60 kPa [28,29]. Therefore, 40 kPa was selected as the baseline, with 20 kPa and 60 kPa chosen for comparison. Concerning loading stress, subgrade dynamic stress is influenced by depth and traffic loads, and under heavy load conditions, it generally does not exceed 100 kPa [30]. Accordingly, three levels of 60, 80, and 100 kPa were adopted, with 80 kPa representing the typical condition. For the loading model, the loading frequency was set as 1 Hz, and each loading cycle consisted of a 0.2 s half-sine loading pulse followed by a 0.8 s rest period. The schematic diagram of the loading waveform is shown in Figure 2. Similar loading modes have been used in previous dynamic triaxial studies of subgrade soils and are consistent with the loading form recommended in NCHRP 1-28A [31]. In addition, previous studies on permanent deformation of subgrade soils have commonly used 10,000 loading cycles as a termination condition or representative loading level for evaluating cumulative deformation under repeated traffic loading [32]. The dynamic triaxial test program is summarized in Table 1, and the overall experimental procedure is illustrated in Figure 3.

## 3. Results and Discussion

### 3.1. Effect of Stress State

**Figure 4:**
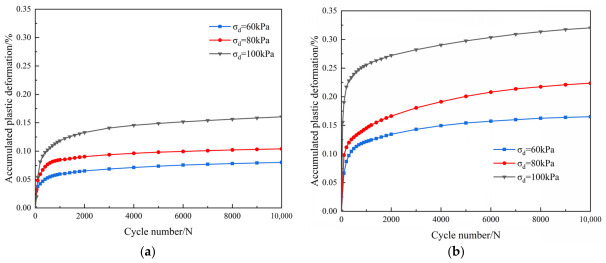
Accumulated plastic deformation curves under different loading stress levels for specimens subjected to 0 and 3 freeze–thaw cycles. (**a**) *N_FT_* = 0 under different loading stress levels. (**b**) *N_FT_* = 3 under different loading stress levels.

According to the experimental program, dynamic triaxial tests on accumulative plastic deformation were conducted for silty sand specimens subjected to *N_FT_* = 0 and *N_FT_* = 3. The deformation evolution curves under loading stresses of 60, 80, and 100 kPa are shown in Figure 4. The results indicate that higher loading stress leads to a greater ultimate value of accumulated plastic deformation. In addition, specimens subjected to freeze–thaw cycles exhibit significantly larger deformation than those without freeze–thaw treatment. At the highest loading stress of 100 kPa, both types of specimens show characteristics of plastic creep, indicating that substantial deformation potential remains with increasing numbers of loading cycles.

Under a confining pressure of 40 kPa, the ultimate deformation values of specimens without freeze–thaw cycles at loading stresses of 60, 80, and 100 kPa are 0.08%, 0.10%, and 0.16%, respectively, whereas the corresponding values for specimens subjected to three freeze–thaw cycles are 0.17%, 0.22%, and 0.32%. When the loading stress increases from 60 kPa to 80 kPa and 100 kPa, the increments for specimens without freeze–thaw cycles are 30% and 100%, respectively, while those for specimens with three freeze–thaw cycles are 35% and 94%. Furthermore, the ultimate deformation of specimens subjected to three freeze–thaw cycles is approximately 2.01–2.14 times that of unfrozen specimens, indicating that freeze–thaw action significantly weakens the deformation resistance of the material. These results demonstrate that a higher loading stress, especially 100 kPa, accelerates deformation accumulation and increases the possibility of plastic creep.

**Figure 5:**
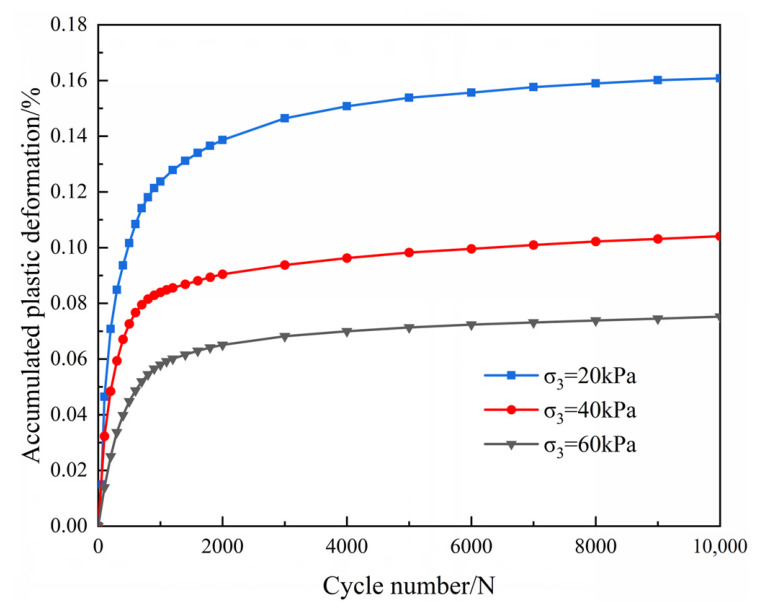
Accumulated plastic deformation curves under different confining pressures for specimens subjected to *N_FT_* = 0.

To investigate the effect of confining pressure on the accumulative plastic deformation of silty sand fill, tests were conducted on specimens that had not undergone freeze–thaw cycles under different confining pressure conditions. The results are shown in Figure 5.

As the loading cycle number rises, the accumulated plastic deformation progressively increases. The development is rapid at the initial stage, with a sharp rise observed within the first 1000 cycles. By approximately 2000 cycles, about 70–80% of the total deformation has already occurred, after which the growth rate gradually slows down. At a loading stress of 80 kPa, higher confining pressure leads to lower accumulated deformation. The ultimate deformation values are 0.16%, 0.10%, and 0.07% under confining pressures of 20, 40, and 60 kPa, respectively. The deformation behavior also varies with confining pressure. Specimens show plastic shakedown at 40 kPa and 60 kPa. At 20 kPa, a plastic creep response is observed, which suggests a potential risk of failure under continued loading. The reduction in deformation with increasing confining pressure is mainly attributed to enhanced lateral confinement, which limits particle movement. In contrast, low confining pressure allows stress concentration to develop more easily within the soil structure. This condition promotes structural damage and leads to greater deformation during subsequent loading.

### 3.2. Effect of Moisture Condition

**Figure 6:**
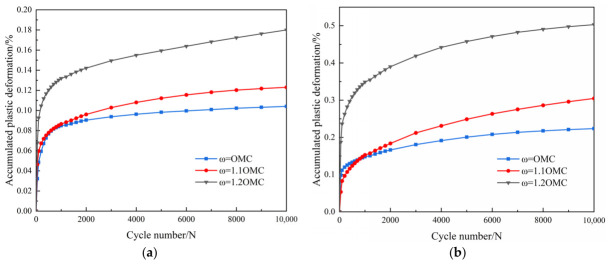
Accumulated plastic deformation curves under different moisture contents for specimens subjected to *N_FT_* = 0 and *N_FT_* = 3. (**a**) Accumulated plastic deformation curves under different moisture contents (*N_FT_* = 0). (**b**) Accumulated plastic deformation curves under different moisture contents (*N_FT_* = 3).

According to the test scheme, dynamic triaxial tests on accumulated plastic deformation were carried out on silty sand specimens with different moisture contents under *N_FT_* = 0 and *N_FT_* = 3. The test results are shown in Figure 6.

As shown in the figure, the deformation development patterns are generally consistent under all test conditions: rapid growth occurs at the initial stage, followed by a gradual reduction in growth rate with increasing cycle number, eventually approaching a stable value.

For both freeze–thaw conditions, the final accumulated plastic deformation increases as moisture content rises, and the largest growth occurs at the highest moisture level. Under *N_FT_* = 0, the corresponding ultimate deformation values for OMC, 1.1 OMC, and 1.2 OMC are 0.10%, 0.12%, and 0.18%, respectively, corresponding to increments of 18.2% and 72.9%. After three freeze–thaw cycles, the deformation values increase to 0.22%, 0.30%, and 0.50%, with corresponding increments of 36.2% and 125.9%. The comparison results show that the final deformation of specimens subjected to *N_FT_* = 3 is significantly greater than that of specimens under *N_FT_* = 0, reaching about 2.15 to 2.79 times higher. Freeze–thaw conditions amplified the influence of moisture content. This indicates that both water content and freeze–thaw effects have significant impacts on the development of deformation. This may be attributed to the increased water content, which thickens the interparticle water films and facilitates relative particle sliding, while the formation of irreversible macropores during freeze–thaw cycles further enlarges the compressible void space, leading to a marked increase in deformation.

### 3.3. Effect of Freeze–Thaw Cycles

**Figure 7:**
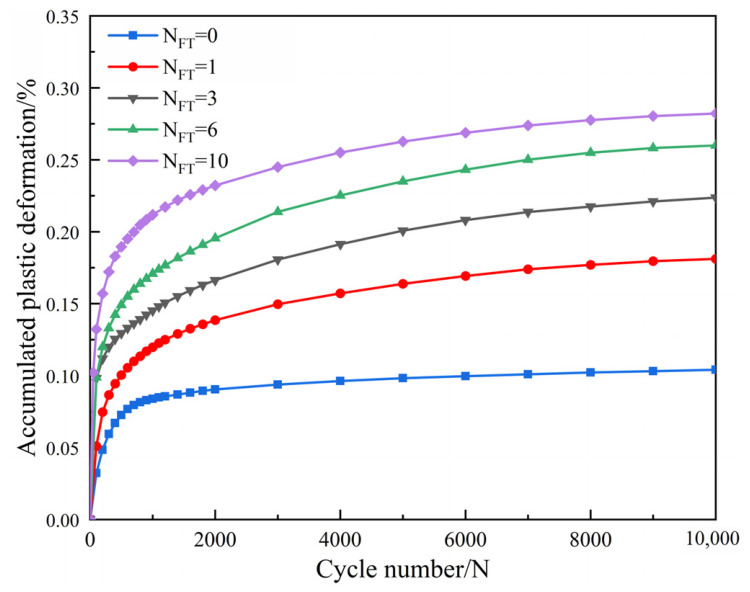
Accumulated plastic deformation curves under different numbers of freeze–thaw cycles (*N_FT_*).

Silty sand specimens were tested under dynamic triaxial conditions with a confining pressure of 40 kPa, an applied loading stress of 80 kPa and the optimum moisture content. The specimens were subjected to *N_FT_* = 0, 1, 3, 6, and 10. The variation in accumulated plastic deformation with loading cycles is shown in Figure 7.

The results show that deformation mainly occurs within the first 2000 loading cycles under all conditions. The specimens with 0 and 1 freeze–thaw cycles become stable after 2000 cycles. The specimens with 3, 6, and 10 freeze–thaw cycles still show obvious deformation after 2000 cycles. This part of deformation accounts for 25.7%, 24.8%, and 26.4% of the total deformation, respectively. These specimens show a trend of plastic creep. This indicates that repeated freeze–thaw cycles increase structural damage and give the specimens a greater deformation capacity. Even after 10,000 cycles, these specimens do not reach a stable state.

After 10,000 loading cycles, the final deformation values of specimens with *N_FT_* = 0, 1, 3, 6, and 10 are 0.10%, 0.18%, 0.22%, 0.26%, and 0.28%, respectively. Compared with the unfrozen specimens, the values are 1.74, 2.15, 2.50, and 2.71 times higher, respectively. Overall, the accumulated plastic deformation increases with the *N_FT_*. The change is most significant after one freeze–thaw cycle. As the *N_FT_* continues to increase, the deformation growth rate gradually decreases and approaches a stable state at 6 and 10 cycles.

The above results indicate that the deformation control of silty sand roadbeds in seasonally frozen regions should consider the combined effects of traffic loading, moisture condition, freeze–thaw cycles, and confinement. During thawing periods, heavy and overloaded vehicles should be controlled to reduce traffic-induced deviatoric stress. In addition, moisture regulation and drainage improvement are important because higher water content amplifies the deformation caused by freeze–thaw cycles. Sufficient lateral confinement, achieved through proper compaction and structural support, may also help restrain particle movement and reduce cumulative plastic deformation under repeated loading.

### 3.4. Accumulated Plastic Deformation Prediction Model for Silty Sand

Numerous researchers have investigated subgrade deformation from multiple perspectives and have proposed various models for predicting accumulated plastic deformation.

Monismith [6] found that the accumulated plastic deformation of silty clay under high-cycle loading deviated from the semi-logarithmic model and proposed an exponential prediction model, which has been widely used and further developed in later studies.

(1) $$\varepsilon_{p}=AN^{b}$$

where A,b are fitting parameters.

Puppala [18] showed that shear action significantly affects the accumulated plastic deformation of subgrade soils. Based on the Monismith model, mean normal stress and octahedral shear stress were introduced to account for confinement and shear effects, respectively, leading to the following prediction model.

(2) $$\varepsilon_{p}=\alpha_{1}N^{\alpha_{2}}{\left(\frac{\sigma_{oct}}{\sigma_{atm}}\right)}^{\alpha_{3}}{\left(\frac{\tau_{oct}}{\sigma_{atm}}\right)}^{\alpha_{4}}$$

where $\varepsilon_{p}$ is the accumulated plastic deformation, and $\alpha_{1},\alpha_{2},\alpha_{3},\alpha_{4}$ are fitting parameters; $\sigma_{oct}$ is mean normal stress; $\sigma_{oct}=\frac{\left(\sigma_{1}+\sigma_{2}+\sigma_{3}\right)}{3}$; $\sigma_{1},\sigma_{2},\sigma_{3}$ denote the major, intermediate, and minor principal stresses, respectively; $\sigma_{atm}$ is the atmospheric pressure, taken as 101.3 kPa. $\tau_{oct}$ is the octahedral shear stress; $\tau_{oct}=\sqrt{{\left(\sigma_{1}-\sigma_{2}\right)}^{2}+{\left(\sigma_{1}-\sigma_{3}\right)}^{2}+{\left(\sigma_{2}-\sigma_{3}\right)}^{2}}/3$; $\sigma_{oct}=\frac{\sqrt{2}}{3}\left(\sigma_{1}-\sigma_{3}\right)$. N is the cyclic loading times of dynamic load.

Based on the above experimental results and test conditions, loading stress, confining pressure, number of freeze–thaw cycles, number of loading cycles, and moisture content were selected as the main influencing factors by extending the Monismith exponential model and the Puppala model. Net mean stress and octahedral shear stress were introduced to distinguish the confinement effect from the shear effect, while the number of freeze–thaw cycles and the water content ratio were incorporated to characterize environmental effects. Based on the experimental data, the prediction model was established as follows:

(3) $$\varepsilon_{p}=0.054{\left(\ln\left(N+1\right)\right)}^{1.214}{\left(\frac{\sigma_{m}}{\sigma_{atm}}\right)}^{-0.693}{\left(\frac{\tau_{oct}}{\sigma_{atm}}\right)}^{1.96}{\left(\frac{\omega }{\omega_{OMC}}\right)}^{4.321}{\left(\ln\left(N_{FT}+e\right)\right)}^{1.239}$$

where $\varepsilon_{p}$ is the accumulated axial deformation, and N is the number of loading cycles. $\sigma_{m}$ is the net stress; $\sigma_{m}=\sigma_{1}+\sigma_{2}+\sigma_{3}-\sigma_{d}$; $\sigma_{d}$ is the loading stress. The $\tau_{oct}$ is the octahedral shear stress; $\omega /\omega_{OMC}$ is the moisture content ratio; the optimum moisture content $\omega_{OMC}$ was set to 14.9%. $N_{FT}$ denotes the number of freeze–thaw cycles. e is the mathematical constant with a value of about 2.718. All coefficients and exponents in the model were determined using least-squares regression based on the measured cumulative plastic deformation data.

**Figure 8:**
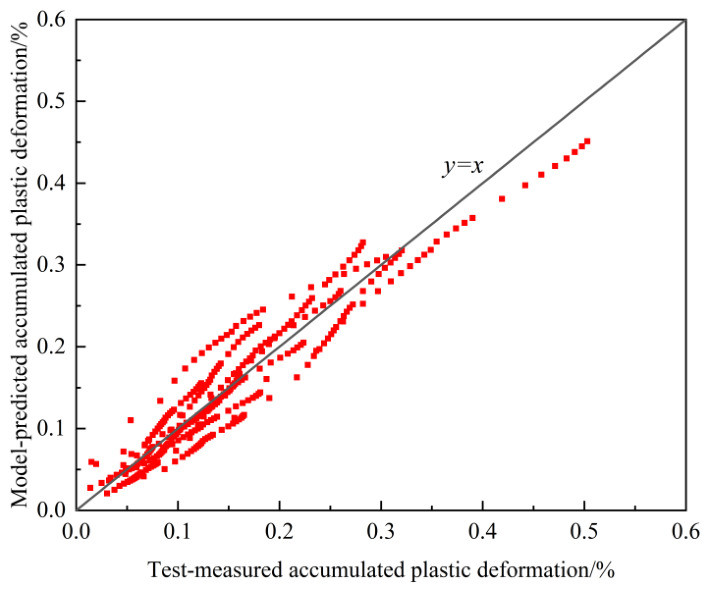
Comparison between model predictions and test results.

The model comprehensively considers stress conditions, cyclic loading, and environmental factors, and is primarily applicable to plastically stable and weakly plastic creep conditions. The classification of these deformation states was interpreted based on the shakedown concept. Previous studies have classified the permanent deformation response of subgrade materials under repeated loading into plastic shakedown, plastic creep, and incremental collapse [33]. In this study, the deformation state was determined according to the development trend of the cumulative plastic deformation curve within 10,000 loading cycles. To validate the proposed predictive model for accumulative plastic deformation of silty sand fill, the model was used to fit the results of the accumulative deformation dynamic triaxial tests. The fitting performed on the experimental data yielded a high correlation coefficient of 0.915, indicating good agreement between the predicted and measured values. A comparison between the measured accumulative plastic deformation and the model predictions is shown in Figure 8, where data points closer to the y = x line indicate a better fit.

**Figure 9:**
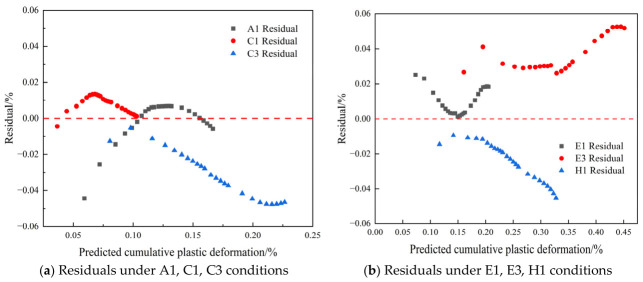
Residual distribution between model predictions and experimental results: (**a**) A1, C1, and C3; (**b**) E1, E3, and H1.

To further evaluate the prediction performance of the proposed model, residual analysis was conducted by calculating the difference between the model-predicted and experimentally measured cumulative axial plastic deformation values. The residual distributions for representative test conditions are shown in Figure 9. Figure 9a presents the residuals under A1, C1, and C3 conditions, while Figure 9b presents those under E1, E3, and H1 conditions. Most residuals are distributed around zero, indicating that the proposed model does not show an obvious systematic bias for the selected test conditions. However, relatively larger residuals are observed under high moisture content and multiple freeze–thaw cycle conditions, namely the C3 and E3 conditions, indicating slightly reduced prediction accuracy under these severe deformation conditions.

**Figure 10:**
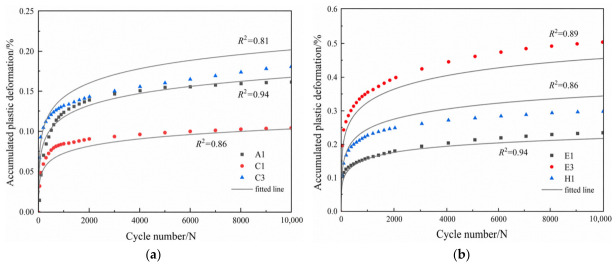
Comparison of typical test conditions and model fitting results. (**a**) A1, C1, and C3 test conditions and model fitting results. (**b**) E1, E3, and H1 test conditions and model fitting results.

The experimental data under representative conditions were compared with the accumulated plastic deformation calculated by the model, as illustrated in Figure 10. In this figure, the symbols represent the measured cumulative plastic deformation, and the solid lines represent the model-fitted results. The coefficient of determination, *R*^2^, shown near each fitted curve was calculated between the measured and fitted values for the corresponding test condition. The comparison shows that the calculated values agree well with the test data overall, and their variation trends are generally consistent: rapid deformation growth occurs within the first 2000 loading cycles, followed by a relatively stable growth stage beyond 2000 cycles.

Overall, the experimental and calculated results showed good agreement at the initial stage of loading. As the number of loading cycles increased, certain differences gradually emerged, but the general trend of change was consistent, and both gradually stabilized. In addition, when the deformation is relatively small, the agreement between the test data and the calculated results is better. For the E3 condition, the discrepancy between the experimental and calculated values after 10,000 cycles is more pronounced than that under other conditions. This may be attributed to the combined effects of higher moisture content and freeze–thaw damage. Condition E3 corresponds to *N_FT_* = 3 and 1.2 OMC. Under this condition, freeze–thaw cycles can disturb the soil structure and promote pore development, while higher moisture content weakens interparticle contact and facilitates particle rearrangement. As a result, the specimen exhibits larger cumulative plastic deformation and a stronger tendency toward weak plastic creep. Since the proposed model is an empirical model calibrated using test conditions, it provides better agreement for small and moderate deformation conditions, while larger deviations may occur under severe deformation conditions with high moisture content and freeze–thaw damage.

**Figure 11:**
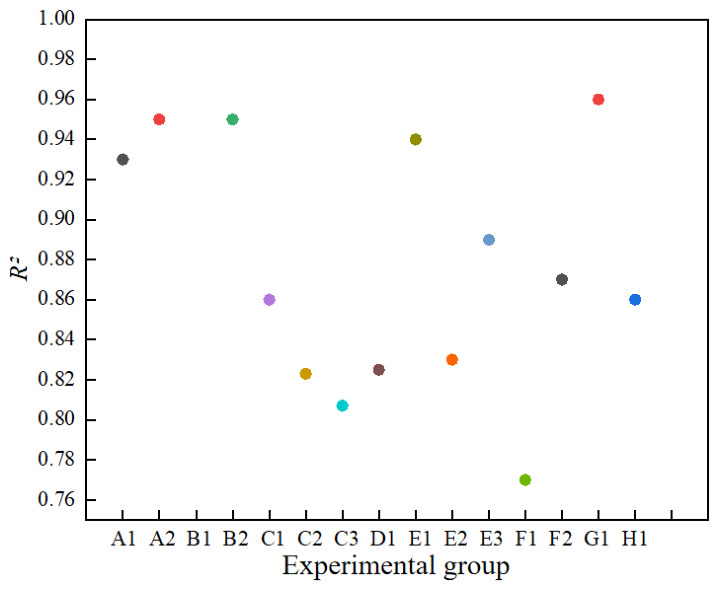
Correlation between experimental data and model predictions under various test conditions.

The fitting performance between the experimental data and the model predictions for all test conditions is shown in Figure 11. The correlation in this figure represents the relationship between the measured cumulative axial plastic deformation and the model-predicted cumulative axial plastic deformation under each test condition. The coefficient of determination, *R*^2^, was calculated based on the measured and predicted values for each condition to evaluate the goodness of fit. A higher *R*^2^ indicates better agreement between the experimental and predicted results. As shown in Figure 11, the *R*^2^ values for most test conditions fall within the range of 0.80–0.95, indicating that the proposed model can reasonably capture the cumulative deformation trend of silty sand under most test conditions. However, it should be noted that the proposed model is an empirical model calibrated using the experimental data obtained in this study. These data were derived from a single soil type and a limited number of deformation curves. Therefore, the model may still have certain limitations when applied to other soil types, wider freeze–thaw cycle ranges, different loading frequencies, or more severe deformation states. Further validation using independent datasets and repeated tests is needed in future studies.

## 4. Conclusions

Based on the experimental program conducted in this study, dynamic triaxial tests were carried out on silty sand specimens to investigate the effects of varying loading stresses, confining pressures, moisture contents, and freeze–thaw cycles (*N_FT_* = 0, 1, 3, 6, and 10) on cumulative plastic deformation. The results presented in Section 3.1, Section 3.2 and Section 3.3 provide a detailed analysis of the deformation behavior under these conditions. The following conclusions were drawn:

(1) The cumulative axial plastic deformation of silty sand develops rapidly during the early stage of cyclic loading and then gradually enters a slower growth stage. Approximately 60–80% of the total deformation occurs within the first 1000 loading cycles, indicating that the initial loading stage is critical for deformation accumulation. Overall, the deformation behavior is mainly characterized by plastic stability or weak plastic creep.
(2) The stress state significantly affects the cumulative plastic deformation of silty sand. Higher loading stress promotes deformation accumulation, whereas higher confining pressure suppresses it. When the loading stress increased from 60 kPa to 100 kPa, the final deformation increased from 0.08% to 0.16% for unfrozen specimens and from 0.17% to 0.32% for specimens subjected to three freeze–thaw cycles. In contrast, when the confining pressure increased from 20 kPa to 60 kPa, the final deformation decreased from 0.16% to 0.07%. This indicates that loading stress accelerates plastic deformation, while confining pressure restrains particle movement and improves deformation resistance.
(3) Freeze–thaw cycles and moisture content jointly weaken the deformation resistance of silty sand. Under the same stress and moisture conditions, the final deformation after three freeze–thaw cycles was approximately 2.01–2.14 times that of unfrozen specimens. As the number of freeze–thaw cycles increased from 0 to 10, the final deformation increased from 0.10% to 0.28%, but the growth rate gradually decreased, indicating that freeze–thaw deterioration is more pronounced during the initial cycles. In addition, moisture content further amplified the deformation response. Under *N_FT_* = 3, the final deformation increased from 0.22% at OMC to 0.50% at 1.2OMC.
(4) A cumulative plastic deformation prediction model was developed by incorporating loading cycles, net mean stress, octahedral shear stress, moisture content ratio, and freeze–thaw cycles. The model achieved a coefficient of determination of *R*^2^ = 0.915, indicating that it can reasonably describe the deformation development of silty sand under plastic stability and weak plastic creep conditions. In addition, residual analysis showed that most residuals were distributed around zero, suggesting that the model did not exhibit an obvious systematic bias under the selected test conditions. However, the model is mainly applicable to the silty sand tested in this study and the selected ranges of loading stress, confining pressure, moisture content, freeze–thaw cycles, and loading frequency. In addition, the current experimental program was limited to a single soil type, a single loading frequency, freeze–thaw cycles up to *N_FT_* = 10, and limited repeated specimens for each condition. Future studies should further verify the model using different soil types, wider freeze–thaw cycle ranges, multiple loading frequencies, repeated specimens for each condition, and independent validation datasets.

## Figures and Tables

**Table 1:** The dynamic triaxial test program.

Group	Confining Pressure	*N_FT_*	Loading Stress	Moisture Condition	Number of Loading Cycles
A1, A2	20, 60	0	80	OMC	10,000
B1, B2	40	0	60, 100	OMC	
C1, C2, C3	40	0	80	OMC, 1.1 OMC, 1.2 OMC	
D1	40	1	80	OMC	
E1, E2, E3	40	3	80	OMC, 1.1 OMC, 1.2 OMC	
F1, F2	40	3	60, 100	OMC	
G1	40	6	80	OMC	
H1	40	10	80	OMC	

## Data Availability

The original contributions presented in this study are included in the article. Further inquiries can be directed to the corresponding author.

## Nomenclature

Symbol/Acronym	Definition	Unit
$\varepsilon_{p}$	Cumulative axial plastic deformation	%
N	Number of loading cycles	-
$N_{FT}$	Number of freeze–thaw cycles	-
$\sigma_{m}$	Net stress term used to characterize confinement effect in the proposed model	kPa
$\sigma_{oct}$	Mean normal stress	kPa
$\sigma_{d}$	Cyclic loading stress	kPa
$\sigma_{atm}$	Atmospheric pressure, taken as 101.3 kPa	kPa
$\tau_{oct}$	Octahedral shear stress	kPa
ω	Moisture content	%
$\omega_{OMC}$	Optimum moisture content	%
e	Euler’s number	-
